# Comprehensive review of the evidence regarding the effectiveness of community–based primary health care in improving maternal, neonatal and child health: 3. neonatal health findings

**DOI:** 10.7189/jogh.07.010903

**Published:** 2017-06

**Authors:** Emma Sacks, Paul A Freeman, Kwame Sakyi, Mary Carol Jennings, Bahie M Rassekh, Sundeep Gupta, Henry B Perry

**Affiliations:** 1 Department of International Health, Johns Hopkins Bloomberg School of Public Health, Baltimore, Maryland, USA; 2 Independent Consultant, Seattle, Washington, USA; 3 Department of Global Health, University of Washington, Seattle, Washington, USA; 4 The World Bank, Washington, District of Columbia, USA; 5 Medical Epidemiologist, Lusaka, Zambia

## Abstract

**Background:**

As the number of deaths among children younger than 5 years of age continues to decline globally through programs to address the health of older infants, neonatal mortality is becoming an increasingly large proportion of under–5 deaths. Lack of access to safe delivery care, emergency obstetric care and postnatal care continue to be challenges for reducing neonatal mortality. This article reviews the available evidence regarding the effectiveness of community–based primary health care (CBPHC) and common components of programs aiming to improve health during the first 28 days of life.

**Methods:**

A database comprising evidence of the effectiveness of projects, programs and field research studies (referred to collectively as projects) in improving maternal, neonatal and child health through CBPHC has been assembled and described elsewhere in this series. From this larger database (N = 548), a subset was created from assessments specifically relating to newborn health (N = 93). Assessments were excluded if the primary project beneficiaries were more than 28 days of age, or if the assessment did not identify one of the following outcomes related to neonatal health: changes in knowledge about newborn illness, care seeking for newborn illness, utilization of postnatal care, nutritional status of neonates, neonatal morbidity, or neonatal mortality. Descriptive analyses were conducted based on study type and outcome variables. An equity assessment was also conducted on the articles included in the neonatal subset.

**Results:**

There is strong evidence that CBPHC can be effective in improving neonatal health, and we present information about the common characteristics shared by effective programs. For projects that reported on health outcomes, twice as many reported an improvement in neonatal health as did those that reported no effect; only one study demonstrated a negative effect. Of those with the strongest experimental study design, almost three–quarters reported beneficial neonatal health outcomes. Many of the neonatal projects assessed in our database utilized community health workers (CHWs), home visits, and participatory women’s groups. Several of the interventions used in these projects focused on health education (recognition of danger signs), and promotion of and support for exclusive breastfeeding (sometimes, but not always, including early breastfeeding). Almost all of the assessments that included a measurable equity component showed that CBPHC produced neonatal health benefits that favored the poorest segment of the project population. However, the studies were quite biased in geographic scope, with more than half conducted in South Asia, and many were pilot studies, rather than projects at scale.

**Conclusions:**

CBPHC can be effectively employed to improve neonatal health in high–mortality, resource–constrained settings. CBPHC is especially important for education and support for pregnant and postpartum mothers and for establishing community–facility linkages to facilitate referrals for obstetrical emergencies; however, the latter will only produce better health outcomes if facilities offer timely, high–quality care. Further research on this topic is needed in Africa and Latin America, as well as in urban and peri–urban areas. Additionally, more assessments are needed of integrated packages of neonatal interventions and of programs at scale.

Despite marked reductions in overall child mortality globally since 1990, 2.7 million live–born infants still die annually during their first month of life [1]. Neonatal mortality is becoming an increasingly large proportion of mortality among children younger than 5 years of age, at present accounting for 45% of under–5 deaths [2]. Approximately 73% of neonatal deaths occur during the first week of life [3], 36% on the first day of life [3] and 32% during the first 6 hours of life [4]. The key causes of death among neonates are complications of preterm birth, intrapartum–related complications (often birth asphyxia), and infections [5]. Given that 51% of births in the least developed countries, 49% of births in sub–Saharan Africa, and 41% of births in South Asia still take place outside of health facilities [1], and the continuing challenges with providing high–quality care in facilities, community–based approaches to improve neonatal health will be essential for the near term to promote healthy home practices and to reach newborns during their birth and soon thereafter when they have a high risk of mortality. Community–based efforts in education, support and referral may be important in settings with high facility delivery rates as well.

Community–based approaches to reducing neonatal mortality are of particular importance in low–income settings where home deliveries are common and access to facility–based care for neonates is limited [2,6,7]. This paper analyzes the findings related to the effectiveness of community–based primary health care (CBPHC) in improving neonatal health using a subset of articles from a database assembled for a broader review of the effectiveness of community–based primary health (CBPHC) in improving child health. It complements other reviews that have been carried out on this topic [7–9]. Projects were assessed by their study design, outcome variables, program components, and reported neonatal health impact.

## METHODS

The methodology for assembling a database of 548 assessments of the effectiveness of CBPHC in improving child health, including the search strategy, has been described elsewhere in this series [10]. In brief, we considered CBPHC to be any activity in which one or more health–related interventions were carried out in the community outside of a health facility. There could also be associated activities that took place in health facilities. The larger study conducted a search of published documents in PubMed, personal sources, and the grey literature for documents that described the implementation of CBPHC and assessed the effect of these projects, programs, or field research studies (described collectively as projects) on mortality, morbidity, nutritional status, or population coverage of an evidence–based intervention. Of 4276 articles identified for screening via PubMed, 433 qualified for the review. In addition, 115 reports were identified from the grey literature and elsewhere, yielding a total of 548 neonatal and child health assessments included in the review. Two reviewers independently extracted information about the assessment and a third independent reviewer resolved any differences. The data were transferred to an electronic database using EPI INFO version 3.5.4 (US Centers for Disease Control and Prevention, Atlanta, Georgia, USA).

**Figure 1:**
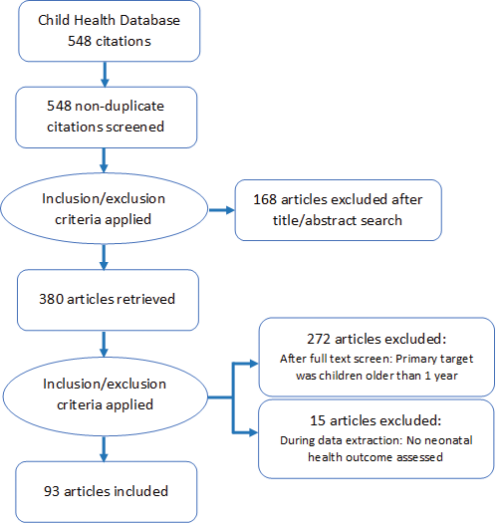
Selection of assessments for inclusion in the neonatal health review.

Starting with the child health data set, assessments were selected for the analysis of neonatal health in a three–stage process (**Figure 1**). In the first stage, articles were selected that had been coded with relevant interventions pertaining to neonates. These interventions, as defined on the data extraction form, were: neonatal/perinatal health; breastfeeding; child weight/height (including birth weight); immunizations; diarrhea treatment; pneumonia treatment; malaria prevention; malaria treatment; Integrated Management of Childhood Illness (IMCI); prevention of mother–to–child transmission of HIV; neonatal tetanus prevention; neonatal tetanus treatment; congenital syphilis prevention; congenital syphilis treatment; and primary health care. This yielded 380 articles.

In the second stage, titles and abstracts of these 380 articles were reviewed. Articles were then excluded if the target population was not infants under age one. This yielded 108 articles. Further exclusions were made if the article did not have an outcome directly related to neonatal health (knowledge about newborn illness, care seeking for newborn illness, utilization of postnatal care, or a neonatal health outcome related to nutritional status, morbidity or mortality). The final database for this sub–analysis included 93 articles. Articles were coded by the primary and secondary health condition addressed, the outcome variables, and categorized by the type and strength of study design.

All study designs were included, but were separated into three categories: randomized controlled trials (RCTs); non–randomized controlled trials; and observational and other non–experimental designs. We conducted descriptive analyses on the data set to present the proportion of beneficial health outcomes within each category. A table of only the RCTs is presented in Table S1 of **Online Supplementary Document(Online Supplementary Document)**.

In this paper, when assessments selected for this analysis are specifically cited, we cite them with the first author’s last name and year of publication, with the reference number in brackets with a prefix S. The full reference can be obtained from Appendix S2 in **Online Supplementary Document(Online Supplementary Document)** where the full references for all the 93 assessments selected for the analysis in this paper can be located.

The term community health worker (CHW) is used here to refer to any community–level actor who receives training from the project or the broader health system/health program to assist in the activities of the project. We do not provide any further specification here regarding length of training, level of compensation (if any), formal recognition by the ministry of health, or other descriptive characteristics of CHWs, as they varied widely among the included assessments, although we recognize that this is an important dimension of these projects.

## RESULTS

### Description of database

**Figure 2:**
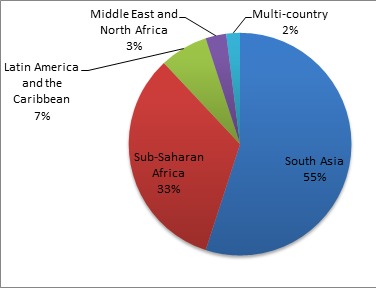
Regions of the world where projects were implemented whose assessments are in the neonatal database (n = 93).

As shown in **Figure 2**, South Asia was far more represented than Africa or Latin America for assessments of the effectiveness of CBPHC in improving neonatal health. The country with the most reported assessments was India (with 16), followed by Bangladesh (12), Nepal (12) and Pakistan (6). Brazil had 4 assessments; Ghana, Kenya, Tanzania and Uganda, each had 3. Two assessments were of projects in more than one country: one implemented in 10 African countries and one in four countries in sub–Saharan Africa and South Asia.

Most of the 93 assessments in our analysis were of projects that focused on a set of communities (n = 36) or a district (n = 42). Very few studies (n = 10) were at the provincial, national or multinational level, and 5 projects were implemented in one community only. Overwhelmingly, the projects were in rural areas (n = 67), although some were in urban (n = 19) or peri–urban areas (n = 7). Projects were mostly implemented by CHWs (n = 61), and many utilized ministry of health staff (n = 37), local field researchers (n = 26) and local community members (n = 27); these categories were not mutually exclusive and there are many projects using paid or volunteer CHWs who were a formal part of ministry of health services.

### Interventions implemented

Three–quarters (76%) of the 93 assessments identified for this review described projects that implemented what were classified in the data extraction process as “neonatal/perinatal health” interventions. Almost one–third of the assessments (38%) described a breastfeeding intervention, and one–quarter (24%) described an intervention that focused on the prevention of low birth weight or the care of low–birth weight infants. Other common activities carried out by these projects included general primary health care, immunizations, micronutrient distribution, malaria prevention or treatment, tetanus prevention, pneumonia treatment, and tetanus prevention; no studies addressed pneumonia prevention or tetanus treatment (**Table 1**).

**Table 1:** Interventions reported in assessments of community–based primary health care in improving neonatal health

Intervention	Number of assessments in review*	Percentage (n = 93)
General promotion of improved neonatal health	67	72.0
Promotion of breastfeeding during the neonatal period	33	35.5
Promotion of improved weight among neonates (including birth weight)	21	22.6
Primary health care	15	16.1
Integrated Management of Childhood Illness (IMCI)	14	15.1
Diarrhea treatment	12	12.9
Malaria treatment	12	12.9
Immunizations	11	11.8
Malaria prevention	7	7.5
Neonatal tetanus prevention	7	7.5
Pneumonia treatment	7	7.5
HIV/AIDS (prevention of mother–to–child transmission of HIV)	5	5.4
Congenital syphilis prevention	1	1.1
Congenital syphilis treatment	1	1.1

*The column sums to more than 93 since many assessments described multiple interventions.

Projects were generally implemented over a relatively short timeline. One–quarter (24%) of the assessments were implemented for less than one year, and another three–quarters (72%) were implemented for between one to five years. Fewer than 5% of the projects in the review were implemented for more than five years.

### Outcomes

The assessments utilized a range of methodologies. Almost half (46%) were randomized controlled trials (RCTs), and another 15% were quasi–experimental (non–randomized, controlled) trials. A fifth of the projects (21%) used an uncontrolled before–after study design, and a tenth (9%) used a descriptive study design. Other study designs less commonly used were case–control and cross–sectional studies. Table S1 in **Online Supplementary Document(Online Supplementary Document)** provides a summary of the RCT assessments.

Among the 93 assessments included in our analysis, 45 separate indicators were measured. **Table 2** and **Table 3** list these and classify them in terms of the Donabedian scheme [11] of input, process, output, outcome and impact indicators and also in terms of the type of outcome. Outcomes were classified as either: (1) a significant positive effect, or (2) no significant effect or (3) a significant negative effect. Positive or negative effects were all statistically significant (*P* ≤ 0.05). No significant effects were those in which statistical testing demonstrated a difference that was not statistically significant (*P* > 0.05), or significance testing was not performed. **Table 2** and **Table 3** provide an analysis of effectiveness in terms of one or more of the types of health indicators that were used in selecting assessments for inclusion in the review by specific health outcome or process/output indicator. A few process/output indicators shown in **Table 3** did not meet the criteria for inclusion in the review (eg, knowledge measures, quality of care measures, care seeking for neonatal illness, participation in group activities, or birth preparedness) but they were measured as part of project assessments along with other health outcome indicators that did qualify, so we have included them in **Table 3**.

**Table 2:** Assessments of community–based primary health care projects that document improvements in neonatal health as defined by health outcome and health impact indicators*

Outcome measure	Assessment methodology with findings						Total
**Randomized controlled assessments**		**Non–randomized controlled assessments**		**Observational (mostly pre/post intervention) assessments**	
**Positive effect (n = 31)**	**No significant or negative effect (n = 12)**	**Positive effect (n = 8)**	**No significant or negative effect (n = 2)**	**Positive effect (n = 13)**	**No significant or negative effect (n = 7)**
**Nutritional status:**							
Birth weight/low birth weight	Christian 2003 [S23]	Larocque 2006 [S46]				Ahrari 2006 [S2]; Tielsch 2008 [S82]	4
Small–for–gestational age		Christian 2003 [S23]					1
Preterm birth	Christian 2003 [S23]						1
**Morbidity:**							
Neonatal sepsis	Gill 2014 [S34]; Soofi 2012 [S77]						2
Neonatal omphalitis	Mullany 2006 [S53]; Soofi 2012 [S77]						2
HIV mother–to–child transmission/infection rate					Gupta 2013 [S36]; Kagaayi 2005 [S40]	Vogt 2015 [S86]	3
Diarrhea/dysentery	Osendarp 2001 [S61]				el–Rafie 1990 [S31]	Tielsch 2008 [S82]	3
Acute respiratory infection	Datta 1987 [S27]					Tielsch 2008 [S82]	2
**Mortality:**							
Neonatal mortality rate	Bang 2005 [S13]; Baqui 2009 [S14]; Bhutta 2008 [S20]; Bhandari 2013 [S19]; El Arifeen 2012 [S30]; Fottrell 2013 [S33]; Kumar 2008 [S45]; Lewycka 2013 [S47]; Manandhar 2014 [S50]; Perry 2006 [S64]; Persson 2013 [S66]; Rahman 1982 [S68]; Tielsch 2007 [S81]; Tripathy 2010 [S83]	Azad 2010 [S9]; Colbourn 2013 [S24]; Gill 2014 [S34]; Kirkwood 2013 [S44]; More 2012 [S52]; Sloan 2008 [S76]; Soofi 2012 [S77]	Bang 1999 [S12]; Memon 2015 [S51]; Spencer 1987 [S78]	Singh 2014 [S74]	Rana 2011 [S69]		26
Early neonatal mortality rate			Memon 2015 [S51]	Singh 2014 [S74]			2
Perinatal mortality rate	Bang 2005 [S13]; Bhutta 2008 [S20]; Kumar 2008 [S45]; Jokhio 2005 [S39]		Bang 1999 [S12]; Memon 2015 [S51]		Seim 2014 [S72]		7
Early infant mortality rate			Christian 2004 [S22]				1
Infant mortality rate	Lewycka 2015 [S47]; Perry 2006 [S64]; Shankar 2008 [S73]	Benn 2008 [S18]; Sloan 2008 [S76]	Perry 2006 [S64]		Anand 2000 [S5]; Li 2007 [S48]; ASHA–India 2008 [S7]	Becker 1993 [S17]	9
Sepsis–specific case fatality rate					Khanal 2011 [S42]		1
Diarrhea–specific mortality						el–Rafie 1990 [S31]	1
Tetanus–specific mortality rate	Rahman 1982 [S68]	Newell 1996 [S59]			Becker 1993 [S17]; ASHA–India 2008 [S7]; Anand 2000 [S5]		5
Pneumonia–specific mortality rate					Bang 1994 [S11]		1
Low birth weight–specific mortality rate	Sloan 2008 [S76]; Tielsch 2007 [S81]						2
**Total number of assessments**	31	12	8	2	13	7	73

*See Appendix S2 in **Online Supplementary Document(Online Supplementary Document)**.

**Table 3:** Assessments of community–based primary health care projects that document improvements in neonatal health as defined by health process/output indicators*

Process and output measures	Assessment methodology with findings						Total
**Randomized controlled assessments**		**Non–randomized controlled assessments**		**Observational (mostly pre/post intervention) assessments**	
**Positive effect (n = 36)**	**No useful or negative effect (n = 5)**	**Positive effect (n = 28)**	**No useful or negative effect (n = 5)**	**Positive effect (n = 31)**	**No useful or negative effect (n = 5)**
**Newborn care practices:**							
Thermal care	Kumar 2008 [S45]; Findley 2013 [S32]	Sloan 2008 [S76]	Khan 2013 [S41]; Syed 2006 [S79]				5
Colostrum administration	Kumar 2008 [S45]		Khan 2013 [S41]; Memon 2015 [S51]	Malekafzali 2000 [S49]	Vir 2013 [S85]		5
Cord cleansing with chlorhexidine	El Arifeen 2012 [S30]; Mullany 2006 [S53]; Mullany 2013 [S54]; Soofi 2012 [S77]				Orabaton 2015 [S60]		5
Delayed bathing of the newborn within the first six hours after birth	Kumar 2008 [S45]; Penfold 2014 [S63]		Khan 2013 [S41]		Sitrin 2015 [S75]		4
Clean hygiene practices for home delivery	Fottrell 2013 [S33]; Kumar 2008 [S45]; Penfold 2014 [S63]		Memon 2015 [S51]; Khan 2013 [S41]		Parashar 2013 [S62]; Sitrin 2015 [S75]		7
**Knowledge on newborn health:**							
Knowledge of newborn danger signs	Findley 2013 [S32]		Khan 2013 [S41]		Callaghan–Koru 2013 [S21]; Dongre 2009 [S29]		4
Knowledge on early breastfeeding			Malekafzali 2000 [S49]				1
Knowledge on feeding during diarrhea episodes			Malekafzali 2000 [S49]				1
**Feeding practices and micronutrient supplementation:**							
Breastfeeding within the first two hours	Findley 2013 [S32]		Memon 2015 [S51]; Crookston 2000 [S26]; Syed 2006 [S79]	Malekafzali 2000 [S49]	Vir 2013 [S85]	Khan 2013 [S41]	7
Proper feeding during diarrhea episodes				Malekafzali 2000 [S49]			1
Exclusive breastfeeding	Bashour 2008 [S16]; Coutinho 2005 [S25]; Haider 2000 [S37]; Qureshi 2011 [S67]; Rotheram–Borus 2014 [S71]; Kimani–Murage 2015 [S43]; Lewycka 2013 [S47]		Balaluka 2012 [S10]; Crookston 2000 [S26]; Haider 2002 [S38]; Khan 2013 [S41]	Malekafzali 2000 [S49]	Neumann 1993 [S57]; Thiam 1995 [S80]	Khan 2013 [S41]; Neumann 1999 [S57]; Neutzling 1993 [S58]	17
Micronutrient supplementation coverage	Bang 2005 [S13]; Benn 2008 [S18]; Daulaire 1992 [S28]; Osendarp 2001 [S61]; Shankar 2008 [S73]	Christian 2003 [S23]				Tielsch 2008 [S82]	7
**Referral and treatment of health conditions:**							
Receipt of Amoxicillin within 24 h of onset of pneumonia symptoms					Murray 2014 [S55]		1
Referral of sick newborns	Ansah Manu 2014 [S6]	Bhutta 2008 [S20]	Baqui 2008 [S15]				3
Treatment of diarrhea with ORT					Thiam 1995 [S80]		1
**Accuracy of assessments and adherence to protocols:**							
Correct determination of low birth weight and very low birth weight by CHWs					Amano 2015 [S4]		1
Error free management of cases of pneumonia by traditional birth attendants			Perry 2016 [S65]				1
Correct interpretation of growth chart by mothers			Malekafzali 2000 [S49]				1
Detection/identification of sick newborns	Ansah Manu 2014 [S6]		Baqui 2008 [S15]		Rana 2011 [S69]		3
Adherence to protocols for management of LBW and VLBW					Amano 2015 [S4]		1
**Health care utilization and birth preparedness:**							
Antenatal care attendance	Persson 2013 [S66]		Uzondu 2015 [S84]; Baqui 2008 [S15]	Memon 2015 [S51]	Wangalwa 2012 [S88]; AFK– Pakistan 2014 [S1]; Rana 2011 [S69]		7
Delivery in a health facility or by a skilled birth attendant	Bhutta 2008 [S20]; Colbourn 2013 [S24]		Memon 2015 [S51]; Uzondu 2015 [S84]; Khan 2013 [S41]		AFK–Pakistan 2014 [S1]; Awoonor– Williams 2004 [S8]; Gopinath 2011 [S35]; Murray 2014 [S55]; Wangalwa 2012 [S88]		10
Receipt of postnatal care	Findley 2013 [S32]	Bashour 2008 [S16]			AFK–Pakistan 2014 [S1]; Wangalwa 2012 [S88]		4
Care seeking for neonatal illnesses	Bhandari 2013 [S19]; Ansah Manu 2014 [S6]			Ali 2005 [S3]	Murray 2014 [S55]; Nalwadda 2013 [S56]; Dongre 2009 [S29]		6
Immunization coverage	Rahman 1982 [S68]; Findley 2013 [S32]	Bashour 2008 [S16]	Memon 2015 [S51]		Becker 1993 [S17]; Nalwadda 2013 [S56]		6
Participation in group activities					Gopinath 2011 [S35]		1
Birth preparedness	Waiswa 2015 [S87]		Perry 2016 [S65]				2
**Total number of assessments**	37	5	28	6	31	5	111

ORT – oral rehydration therapy, LBW – low birth weight, VLBW – very low birth weight

*See Appendix S2 in **Online Supplementary Document(Online Supplementary Document)**.

Overall, 31 of the 43 measurements of outcomes of randomized controlled assessments that are shown in Table 2 demonstrated positive effects: 2 out of 4 for nutritional status, 6 out of 6 for morbidity, and 24 out of 34 for mortality. Among the 10 measurements among non–randomized controlled assessments (all of which were mortality assessments), 8 out 10 demonstrated positive effects. Among the uncontrolled observational (mostly pre/post intervention) assessments, 13 out of 20 (65%) demonstrated positive effects.

This analysis indicates that, for a range of indicators, between 65–90% of the assessments included in our analysis observed a positive outcome or a favorable health impact. Among the 43 randomized controlled trials (RCTs), 31 (72%) showed a positive outcome and 12 (28%) showed either no effect or (in one case) a negative effect.

Of the 50 non–randomized and observational assessments included in our analysis (mostly pre/post intervention assessments), 13 out of 20 (65%) demonstrated a positive outcome. Similarly, for the health process/output measures shown in **Table 3**, the findings are strongly favorable. 37 out of 42 (88%) measurements among randomized assessments demonstrative positive effects, as did 28 out of 34 (82%) measurements among non–randomized controlled assessments and 31 out of 36 (86%) measurements among observational studies (which were mostly pre/post intervention assessments).

Table S1 in **Online Supplementary Document(Online Supplementary Document)** provides details of the 43 randomized controlled trials included among our assessments.

### Implementation strategies

A more detailed analysis of community–based implementation strategies for improving maternal, neonatal and child health is contained in another article in this series [12]. However, here we mention some of the findings that relate specifically to neonatal health interventions.

Key intervention implementation strategies that were utilized in CBPHC projects that improved neonatal health included: home visitation by CHWs for education in relation to prevention, recognition of danger signs, and early treatment/referral of neonates with serious illnesses; community–based treatment and early referral by CHWs for neonatal sepsis; outreach from health facilities, especially for antenatal care and maternal immunization against neonatal tetanus; and participatory women’s groups (sometimes referred to as support groups) to raise awareness about healthy practices during pregnancy and for the newborn, and to raise awareness of danger signs for which facility–based care should be sought.

**Figure 3:**
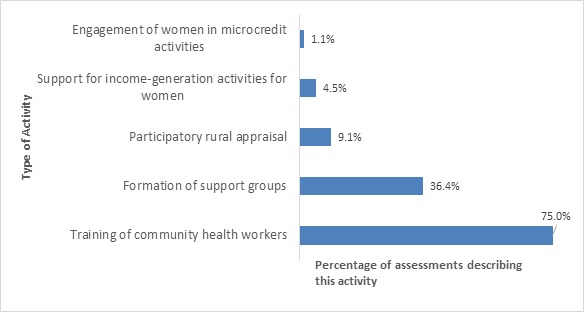
Common associated activities carried out in the implementation of CBPHC projects to improve neonatal health (n = 93). The sum is greater than 100% since some projects had more than one of these activities.

As shown in **Figure 3**, the most common associated implementation strategies were the training of CHWs (carried out in 75% of the projects) and the formation of women’s support groups (present in 36% of the projects).

**Figure 4:**
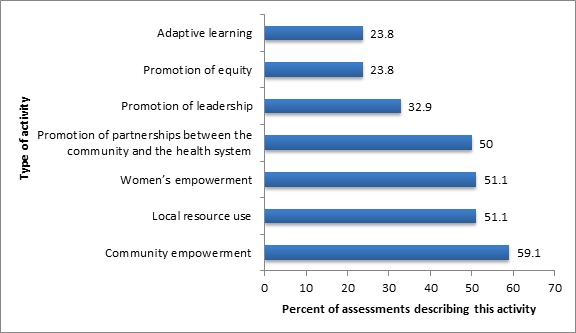
Common associated goals and activities carried out in the implementation of CBPHC projects to improve neonatal health (n = 93). The sum is greater than 100% since many projects employed more than one strategy.

As shown in **Figure 4**, over half of the projects had stated goals and associated activities of promoting women’s or community empowerment, forging links between the community and the health system and promoting local resource use. Less–commonly stated goals and activities were promotion of community leadership, adaptive learning and promotion of equity.

The data extraction form asked reviewers to subjectively judge whether the assessment observed any effect of community participation on health outcome and whether or not the outcome was positive. In 65% (60) of the 93 reports, community participation was reported to have had an effect, and in all of these cases the effect was judged to be positive. In over half (52%) of the 93 reports, the reviewers judged that the linkages between the community and the health system had an impact on health outcomes, and the effect on neonatal health was positive in almost all (93%) of these cases.

### Equity

In terms of coverage, community–based efforts are generally designed to be more equitable than facility–based approaches in reaching those most in need and in improving the health of the most disadvantaged. This arises from the fact that community–based approaches contain strong outreach elements and are often able to reach those who have difficulties in accessing facility–based health care, whether because of distance or socioeconomic challenges such as cost or culture barriers. The equity effects assessed among all the child health projects in our database are described elsewhere [13]. Here, however, we present the findings specific to neonatal health projects.

In total, 8 of the 93 assessments in our neonatal health review examined equity of health outcomes, using different categories of equity (income, geography, etc.). Of the 10 equity assessments reported for these 10 projects, 14 (82%) were considered to be “pro–equitable” (ie, the outcomes were more favorable for the newborns in the most disadvantaged households). For one equity assessment (10%), the outcome was considered to be “equitable” (ie, the outcome was equally favorable in the most disadvantaged and other households), and in only two equity assessment (20%) the outcomes were “inequitable” (ie, the outcomes were less favorable for newborns in the most disadvantaged households compared to other households) (**Table 4**).

**Table 4:** Equity assessments of community–based primary health care in improving neonatal health*

Outcome of assessment	Outcome indicator	Equity category	Reference
**Pro–equitable**	**Mortality**		
	Neonatal mortality rate	Geography	ASHA–India 2008 [S7])
	Neonatal mortality rate	Geography	Bang 1999 [S12]
	Perinatal mortality rate	Geography	Bang 2005 [S13], Bang 1999 [S12]
	**Postnatal care**		
	Postnatal care coverage	Socio–economic status (including education)	Awoonor–Williams 2004 [S8]
	**Skilled birth attendance**		
	Skilled attendant at birth	Socio–economic status (including education)	Awoonor–Williams 2004 [S8]
	**Breastfeeding**		
	Exclusive breastfeeding from birth to 6 mo	Geography	Crookston 2000 [S26]
	Breastfeeding initiation within the first hour of life	Geography	Crookston 2000 [S26]
**Equitable**	**Mortality**		
	Tetanus neonatorum mortality rate	Geography	Newell 1966 [S59]
**Inequitable**	**Mortality**		
	Neonatal morality rate	Socio–economic status	Razzaque 2007 [S70]
	**Breastfeeding**		
	Exclusive breastfeeding from birth to 6 mo	Socio–economic status	Coutinho 2005 [S25]

*See Appendix S2 in **Online Supplementary Document(Online Supplementary Document)**.

## DISCUSSION

Our analysis provides strong evidence that CBPHC can improve neonatal health in low–income settings. Of the studies with strong experimental research designs, over 70% showed a positive neonatal health impact. Although many of these studies were smaller scale pilots or efficacy studies, it demonstrates that CBPHC can be an essential tool where access to facilities is limited and many births take place at home. In these settings, access to antenatal care is often limited; for example, only 49% of pregnant women in sub–Saharan Africa obtain four antenatal care visits [1]. Furthermore, among the 75 countries with the greatest burden of neonatal mortality, the median national coverage of interventions that are important for improving neonatal mortality is quite low: 65% for skilled attendant at delivery, 28% for postnatal visits for newborns, and 50% for early initiation of breastfeeding [14]. Community–based approaches will be essential for the near term in order to achieve universal coverage of health services for these mothers during their delivery and immediately following birth. Even if primary health care services are better developed and facility coverage of antenatal, delivery, and postnatal care increases, CBPHC can continue to make a contribution to improved neonatal health through promotion of healthy household practices and awareness of danger signs for which facility–based care should be sought.

The most common outcome indicators used in the assessments included in our analysis were related to population coverage of postnatal care and exclusive breastfeeding during the neonatal period; mortality was also relatively well–studied. While our review did not include assessments of the quality of implemented interventions or the degree to which projects were implemented under ideal vs more routine conditions (to assess to what degree the assessments were of CBPHC efficacy as opposed to effectiveness), we did summarize the findings by the rigor of the study design and demonstrated that for all levels of methodological rigor, CBPHC approaches appeared to produce favorable outcomes on neonatal health. It is worth noting the importance of assessing and improving the quality of care provided at the time of health contacts between patients and providers, whether they take place in facilities or in homes; however, information on this topic was missing in almost all of the assessments included in our analysis. Further, many of the studies with the strongest designs also had the most intensive support in carrying out the intervention, making it more difficult to judge the effectiveness if scaled up without focused attention or resources.

Our analysis reveals that many of the leading causes of death among children during the first month of life – especially those caused by infection – can be effectively addressed at the community level by CHWs if they have proper training and support. Home–based neonatal care includes promotion of immediate and exclusive breastfeeding, promotion of cleanliness, application of a topical antiseptic (chlorhexidine) to the umbilical cord, prevention of hypothermia, and early diagnosis and referral for treatment of neonatal sepsis. Strong evidence was found for the capacity of CHWs to promote clean delivery, especially in settings where births occur at home and hygiene is poor, to improve neonatal care practices at home, and to identify sick neonates in need of further care and treatment for certain conditions.

Given that many neonatal care projects utilize community health workers (CHWs), it is expected that many interventions can be provided close to or in the home, especially if CHWs live near their patients. Key community–based intervention strategies that were demonstrated to be successful in our analysis include home visitation by CHWs to educate mothers about healthy household practices, danger signs, the importance of early referral and treatment of neonates with danger signs, and outreach by mobile teams from health facilities (especially to provide maternal immunization against neonatal tetanus). Additionally, our analysis identifies the capacity of participatory women’s groups to raise awareness about healthy practices during pregnancy and the postpartum/postnatal period, and to educate about of danger signs for which facility–based care should be sought and the favorable effects of this approach for reducing neonatal mortality. Our equity analysis shows that almost all of the CBPHC interventions for improving newborn health benefit more disadvantaged groups to a greater degree than others.

This study had a number of limitations. The evidence is derived from projects mostly in rural South Asia. Most projects had a relatively short timeline and so we are unable to ascertain if they were successful in the long term. Furthermore, many (but not all) of the projects were implemented in relatively small populations under relatively ideal circumstances in which high–quality training, supervision, and logistical support were assured. So whether similar results can be achieved under more routine condition in larger populations over long periods of time is not known at present.

The large proportion of positive outcomes could be partially due to publication bias. Especially given that all study types were included (such as gray literature reports), there may have been a tendency by organizations to promote their successful work and only publish studies which had a beneficial impact. This study was further limited by the wide range of definitions, indicators and measurements used, which made standardization impossible. We aimed to provide useful categories and definitions, but the variation is wide. For example, it is known that the capacity and competence of CHWs varies widely; further analysis of the details regarding how CHWs were trained and deployed in the projects included in our review were limited. The context in which projects were carried out is also wide: details regarding exactly how the intervention strategies were carried out, and the specific conditions required for them to be effective at scale, go beyond the scope of this analysis. Finally, while this is intended to be a comprehensive review, the field is vast and some studies may not have been included.

The need to accelerate declines in neonatal mortality is readily apparent. In order to achieve universal health coverage and to end preventable neonatal deaths by the year 2030, basic and essential evidence–based neonatal health care interventions will need to reach all mothers and their newborns. Since many countries will not be able to provide universal coverage of essential newborn services by 2030 through facility–based services, progress in reducing neonatal mortality in high–mortality, resource–constrained settings will have to partially depend for the foreseeable future upon strengthening the types of interventions and approaches described here, and on improving timely referral to facilities for newborns with complications. The next step in this process is to test the types of interventions and approaches described here at scale using rigorous operations research methodologies. Further research is also needed in a wider variety of geographic areas, in urban and peri–urban settings, and for longer–term programs.

According to one recently published analysis based on modeling tools [2], immediately scaling up the currently available community–based interventions with evidence of effectiveness for reducing neonatal mortality to reach 90% population coverage would avert an estimated 740 000 neonatal deaths annually (27.4% of the total of 2.7 million neonatal deaths currently occurring each year). Similarly, a separate analysis [15] estimates that 700 000 newborn lives that would be saved if all of the community–based interventions gradually achieved a coverage of 90% over a 5–year period. While CBPHC approaches for reducing the number of stillbirths were not included in this review, there is growing evidence that community–based efforts to improve antenatal care, especially nutrition and malaria prevention, will have effects on the prevalence of stillbirth worldwide [15]. If the interventions that can be provided at primary health care centers and at hospitals but not in the community (eg, full supportive care for preterm newborns or treatment if very serious infection) were able to reach 90% of the neonates who need them, an additional 760 00 neonatal deaths could be averted (170 000 at primary health care centers and 0.59 million at hospitals) [2]. Thus, even though facility–based care is important for improving neonatal health, expanding the coverage of community–based services will also be essential in order to quickly accelerate the decline of neonatal mortality in high–burden countries.

## CONCLUSIONS

The evidence regarding the potential of CBPHC to improve neonatal health in resource–constrained settings is strong. Now there is a need to begin to assemble evidence regarding the effectiveness of implementation of these interventions and strategies at scale. The scaling up of effective community–based interventions will be essential for accelerating progress in reducing neonatal mortality in the near term and for reaching universal coverage of evidence–based interventions for improving neonatal health. Based upon the current evidence, this will require the development and strengthening of a community–based platform involving (1) training and deployment of CHWs to visit homes frequently to promote healthy household behaviors, identification of neonates in need of referral, and utilization of health facilities appropriately, (2) formation and support of participatory women’s groups, and (3) strengthening of outreach services provided by mobile health teams for provision of antenatal and postnatal care. Identifying ways for all newborns to receive the highest quality of care that can be provided in the home will have a sizable impact on neonatal mortality and morbidity worldwide.
