# Supplementary material for: Comprehensive review of the evidence regarding the effectiveness of community–based primary health care in improving maternal, neonatal and child health: 3. neonatal health findings
Source: J Glob Health. 2017 Jun 29;7(1):010903. doi: 10.7189/jogh.07.010903 (PMC5491944; doi:10.7189/jogh.07.010903)
Supplement: Online Supplementary Document [file jogh-07-010903-s001.pdf]

Online Supplementary Document

Sacks et al. Comprehensive review of the evidence regarding the effectiveness of community–based primary health care in improving maternal, neonatal and child health: 3. neonatal health findings

J Glob Health 2017;7:010903

**Table S1. Summaries of studies using randomized control trials (RCTs) to assess neonatal community-based interventions**			
**Outcome measure**	**Author and year of publication**	**Population and study design**	**Results**
**Nutritional status**			
Birth weight/low birth weight	Christian 2003 **S23**	The study sought to determine the effect of micronutrient supplementation on neonatal mortality. Designed as double blind cluster randomized control trial, intervention clusters were assigned to either five micronutrient supplement groups: 1) folic acid alone, 2) folic acid+iron, 3) folic acid+iron+zinc, or 4) multiple micronutrients. Control group received only Vitamin A. 4,130 pregnant women were enrolled in the study. Pregnant women received supplements through sector distributors. The study was conducted in Nepal.	The risk of low birth weight in the folic acid +iron supplements group was 16% lower than that of the Vitamin A only group 16 (RR: 0.84, 95% CI: 0.72-0.99). Compared to Vitamin A only group, the risk of low birth weight in the multiple micronutrients group decreased by 14% (RR: 0.86, 95% CI: 0.74-0.99). Folic iron supplementation alone however had no effect on birth weight. (RR: 1.00, 95% CI:0.88-1.15)
	Larocque 2006 **S46**	Implemented in Peru, the study examined the effect of antenatal mebendazole on low birthweight in a hookworm endemic area. The study enrolled 1042 pregnant women in a double-blind, randomized controlled trial. Women in the intervention group received 500mg of mebendazole and daily iron supplements. Control group received placebo plus iron supplements.	The proportion of low birth weight infants in the intervention and control clusters did not differ (8.1% vs. 8.7%, p=0.755). The proportion of very low birthweight infants however was significantly lower in the intervention than the control clusters (0% vs. 1.5%, respectively; p=0.007).
Small for gestational age	Christian 2003 **S23**	See Christian above	None of the micronutrient supplement combinations reduced small for gestational age. (RR ranged from 0.91 to 1.02, p=0.17)
Preterm birth	Christian 2003 **S23**	See Christian above	Micronutrient supplementation had no effect on preterm birth (RR ranged from 0.99 to 1.08, p=0.77).

Table S1. Summaries of studies using randomized control trials (RCTs) to assess neonatal community-based interventions

Outcome measure	Author and year of publication	Population and study design	Results
**Morbidity**			
Neonatal omphalitis	Mullany 2006 **S53**	The study was a cluster randomized trial to test the effect of CHX on neonatal mortality. 413 communities in Sarlahi, Nepal, were randomly assigned to one of three cord care regimens: 1) cleansing with 4·0% chlorhexidine (CHX) (4,934 infants), 2) cleansing with soap and water (5107), and 3) cleansing dry cord care (5082). The cord care group was the control group.	The risk of omphalitis was reduced by 32%-75% comparing the CHX vs. dry cord care clusters. The effect of CHX was greater in reducing severe omphalitis (Moderate or severe redness, IRR: 0. 0.68 95% CI: 0.58–0.80, Severe redness with pus, IRR: 0.25, 95% CI: 0.12-0.53). Cleansing with soap and water had no effect on moderate to severe omphalitis (IRR1.01, 95% CI: 0.58–1.77).
	Soofi 2012 **S77**	The primary goal of the study was to examine the effect of CHX on neonatal mortality at the community level. The study design was a two-by-two cluster randomized trial and implemented in Sindh Province, Pakistan. 187 clusters, consisting of 9,741 newborn, were assigned to one of four groups: 1) 4% CHX (CHX) solution only, 2) CHX plus hand washing, 3) hand washing only and 4) hand washing with antiseptic (control group).	Compared to the control clusters, the incidence of omphalitis was reduced by 47% in the hand washing plus CHX clusters (RR: 0·53, 95% CI: 0.32–0.88); 37% in the hand washing only clusters (RR: 0.67, 95% CI: 0·48–0.93); and 56% in the CHX only clusters (RR: 0.44, 95% CI: 0.29–0.67).
Diarrhea/dysentery	Osendarp 2001 **S61**	The aim of the study was to test the effect of zinc supplementation during pregnancy on incidence of diarrhea and dysentery in infants at age 6 months. 420 Bangladeshi infants were enrolled in a double-blind, placebo controlled randomized control. In the intervention clusters (with 199 infants), pregnant women took 30mg of zinc beginning at 12 to 16 weeks. The control clusters (221 infants) received placebo.	Among children born low birth weight at birth, at age six months, there was a 16% lower risk of acute diarrhea and 64% lower risk of dysentery in the intervention compared to control clusters (for acute diarrhea: RR: 0.84; 95% CI: 0.72-0.98; for dysentery RR:0.36; 95% CI: 0.25-0.84).

**Table S1. Summaries of studies using randomized control trials (RCTs) to assess neonatal community-based interventions**			
**Outcome measure**	**Author and year of publication**	**Population and study design**	**Results**
Acute respiratory infection	Datta 1987 **S27**	The study examined the feasibility and effect of training primary health care workers on management of acute respiratory infections among children born low birth weight. The study design was a two-stage, cluster randomized design. 21 villages in Havarna, India were assigned to intervention (i.e. training on recognition and treatment of acute respiratory infections) and 16 to control. 199 low birth weight infants were born in the intervention clusters and 211 in the control clusters.	The case fatality (per 100 attacks) of acute respiratory infection among low birth weight neonates was 35% lower in the trained primary health care workers arm than the control arm (8.7 deaths per 100 attacks vs. 24.6 per 100 attacks; although paper does not report 95% CI) (p<0.01)
**Mortality**			
Neonatal mortality rate			
	Azad 2010 **S9**	This stratified, cluster-randomized trial in three rural districts of Bangladesh aimed to assess the effect of facilitator-led support groups, traditional birth attendant training and health system strengthening on neonatal mortality over the 3 year study period. Outcomes were monitored for 36,113 births.	Adjusted NMR in intervention clusters was lower than comparison areas (33.9 per 1000 live births vs. 36.5 per 1000 live births, respectively); (RR: 0.93; 95% CI: 0.80-1.09).
	Bang 2005 **S13**	This field trial was conducted in Gadchiroli, India. 39 villages were assigned to an intervention group and 47 to a control group. The goal of the study was to determine the effect of a home-based neonatal health package delivered through traditional birth attendants on neonatal mortality. The control group received standard care. The intervention included training of birth attendants and treatment of pneumonia and minor illness.	Neonatal mortality rate (NMR) in the intervention area was reduced by 70% (% Change: 70, 95% CI: 59-81) and the absolute difference in NMR between the intervention and comparison area was 44 deaths per 1000 live births.
	Baqui 2009 **S14**	The trial assessed effect of timing of first pgostnatal home visit by lady health workers on neonatal mortality. 24 administrative units in Sylhet, Bangladesh were randomly assigned to either home care, community care, or comparison arm. Pregnant women in the home care	The risk of neonatal mortality was reduced by 67% in those who received a visit on day one than those who received no visit, among those who survived the first day (Adjusted hazard ratio (HR): 0.33, 95% CI: 0.23-0.46).

Table S1. Summaries of studies using randomized control trials (RCTs) to assess neonatal community-based interventions			
**Outcome measure**	**Author and year of publication**	**Population and study design**	**Results**
		intervention group received visits from female health workers on day 1, 3, and 7 postpartum. Analysis was restricted to the home care group and included 9,211 live births were enrolled in the study.	Among those who survived the second day, the risk of neonatal mortality was reduced by 64% in those who received a visit on the second day compared to those who did not receive a visit. (HR:0.36, 95% CI: 0.23-0.55)
	Bhutta 2008 **S20**	The study examined the feasibility of using lady health workers and traditional birth attendants in rural Pakistan to deliver a package of community-based intervention for improving perinatal care. A total of 315 villages, spread across eight village clusters in Pakistan were randomly assigned to intervention and comparison groups (four cluster per group). The intervention package included provision of essential maternal and newborn care practices, community education group sessions, and partnership between lady health workers and local traditional attendants at delivery. The total population of the villages were 138,000.	In the intervention clusters, neonatal mortality rate declined by 28% compared to control clusters (Mantel-Haenszel RR: 0.72, 95% CI: 0.56–0.91).
	Bhandari 2013 **S19**	The trial assessed the effect of an integrated management of neonatal and childhood illness (IMNCI) on neonatal and infant mortality. Intervention included community health worker training and health system strengthening. Implemented in India, 18 clusters were assigned to either intervention or control. 29,667 and 30,813 births occurred in the intervention and control, respectively.	The risk of neonatal mortality was 20% lower in the intervention compared to the control clusters (HR: 0.80; 95% CI: 0.68-0.93).
	Colbourn 2013 **S24**	The study evaluated the effect of participation in a women's participatory group intervention (CI) at a community level combined with facility-based quality improvement intervention (FI) on maternal, perinatal and neonatal mortality. The study design was a two-by-two factorial cluster randomized controlled trial. Clusters were assigned to one of four groups: control (17 clusters, 4912 births), FI only (15 clusters, 5335 births), CI only (15 clusters, 5080 live	The odds of neonatal mortality in the combined FI and CI intervention group was 22% lower than that of the control clusters (OR: 0.78, 95% CI: 0.60-1.01).

**Table S1. Summaries of studies using randomized control trials (RCTs) to assess neonatal community-based interventions**			
**Outcome measure**	**Author and year of publication**	**Population and study design**	**Results**
		births), and FI and CI (14 clusters, 5249 live births). The study was implemented in three districts in Malawi.	
	El-Arifeen 2012 **S30**	The objective of the study was to test the effect of cord cleansing with CHX on neonatal mortality in rural Sylhet, Bangladesh. The study used parallel cluster randomized trial design. 29,760 newborn infants born were randomized into three groups: multiple-cleansing, single cleansing, and dry cord care (control arm). Each intervention was delivered by a community health worker.	Neonatal mortality in the single-cleansing group was reduced by 20% (RR: 0·80; 95% CI: 0·65–0·98) compared to the dry cord care group. There was no effect of multiple cleansing on neonatal mortality. (RR: 0.94; 95% CI: 0.78-1.14).
	Fottrell 2013 **S33**	The study explored the effect of coverage of participatory women groups on neonatal mortality, using a cluster-randomized control design. 18 villages in Bangladesh were randomized to intervention (women's groups) and control groups. The intervention included 840 women's groups and 17,940 live births.	The risk of neonatal mortality in the intervention areas was 38% lower than that of the control clusters (RR: 0.62; 95% CI: 0.43-0.89).
	Kirkwood 2013 **S44**	Implemented in Ghana, and designed as cluster randomized trial, 49 zones were assigned to control clusters and 49 to an intervention cluster (home-visits by a community based surveillance volunteer during pregnancy and 3 days postpartum). A total of 16,329 live births were included in the study. The goal of the study was to determine if home-visits during pregnancy and postpartum contribute to reduction in neonatal mortality.	Home visits by community-based surveillance volunteers had no effect on neonatal mortality (RR: 0.78, 95% CI:0.56–1.08)
	Kumar 2008 **S45**	The objective of this study was to determine the impact of a community-based behavior change management on neonatal mortality. The study design was a cluster-randomized control trial. 39 villages in Uttar Pradesh, India, were assigned to one of these three groups: 1) essential newborn care practices, or 3) liquid crystal hypothermia indicator (ThermoSpot) plus essential newborn care practices 2) control (usual government and non-government services).	The risk of neonatal mortality in the clusters that received the ThermoSpot intervention was 52% lower than that of the control clusters (RR: 0.48, 95% CI: 0.35-0.66). The clusters that received essential newborn care alone had a 54% lower risk of neonatal mortality compared to that of control clusters (RR: 0.46, 95% CI: 0.35-0.60).

**Table S1. Summaries of studies using randomized control trials (RCTs) to assess neonatal community-based interventions**			
**Outcome measure**	**Author and year of publication**	**Population and study design**	**Results**
	Lewycka 2013 **S47**	This was a factorial, cluster-randomized trial of 48 clusters in rural Malawi of community groups and peer counselor home visits. Community groups used Community Action Cycles focused on maternal and child health. Outcomes were monitored on mortality and exclusive breastfeeding for 26,262 births.	Stratified, adjusted analysis shows a 41% reduction in neonatal mortality (OR: 0.59; CI: 0.40-0.86) in areas with community groups only. NMR was not assessed for areas with peer counselor visits only.
	Manandhar 2004 **S50**	This was a randomized trial with pair-matched clusters of facilitator-led women's groups focused on "perinatal problems" in rural Nepal. Birth outcomes were monitored in a cohort of 28,931 women over 3 years, of whom 8% participated in groups.	The neonatal mortality rate was lower in intervention than comparison clusters (26.2 per 1000 live births vs. 36.9 per 1000 live births) (adjusted OR: 0.70; CI: 0.53-0.94). Stillbirth rates were similar in both clusters.
	Memon 2015 **S51**	The study implemented a community-based perinatal and newborn care intervention in Gilget-Baltistan Province, Pakistan. It was designed as a quasi-experimental study. 20 villages with 16,802 households were assigned to the intervention group and 20 villages with 18,659 households to the control group. The intervention was a preventive newborn care package (early initiation of breastfeeding, recognition of danger sings, and delayed bathing).	The odds of perinatal mortality comparing the intervention to control site was reduced by 38% (OR: 0.62, 95% CI: 0.56-0.69). There was a 42% reduction in the odds of neonatal mortality in the intervention compared to the control villages (OR: 0.58, 95% CI: 0.48-0.68).
	More 2012 **S52**	The study was a cluster-randomized trial of facilitator-supported 24 intervention and 24 comparison settlements in urban Mumbai, India. Vital statistics were recorded for 18,197 infants over 3 years.	The neonatal mortality rate higher in the intervention arm (OR: 1.48, 95% CI: 1.06-2.08).
	Mullany 2006 **S53**	See Mullany 2006 above	There was no statistically significant effect of CHX on neonatal mortality, though there was a 24% reduction comparing the CHX group to the dry cord care group (RR:0.76, 95% CI: 0.55-1.04)

**Table S1. Summaries of studies using randomized control trials (RCTs) to assess neonatal community-based interventions**			
**Outcome measure**	**Author and year of publication**	**Population and study design**	**Results**
	Persson 2013 **S66**	The study tested the effect of local community groups that included local health care staff and politicians on maternal and newborn indicators, using a cluster randomized control design. 44 communes in Quan Ninh province, Vietnam, were randomly allocated to an intervention group and 46 to a control group. The analysis included 22,561 births.	The odds of neonatal mortality rate was reduced by 49% in the intervention communes compared to the control communes (OR: 0.51, 95% CI: 0.30-0.89).
	Tielsch 2007 **S81**	The study was a community-based placebo controlled cluster randomized trial. The objective was to test the effect of cleansing neonates with wipes that contained CHX on neonatal mortality. The study was carried out in Sarlahi District, Nepal. A total of 8,650 and 8,880 live births were born in the intervention and control clusters, respectively. Newborns in the intervention clusters were cleansed with wipes that contained CHX.	Cleansing with infant wipes with CHX had no effect on neonatal mortality (RR: 0.89, 95% CI: 0.72-1.10).
	Sloan 2008 **S76**	The study examined the effect of community-based kangaroo mother care on neonatal mortality in rural Bangladesh. As a cluster randomized control trial, 21 divisions were assigned to a community-based kangaroo care intervention and 21 to a control group. The study enrolled 4165 live births. Each newborn was followed for 30-45 days.	Community-based kangaroo mother care had no effect on neonatal mortality (OR: 1.06, 95% CI: 0.76–1.47).
	Soofi 2012 **S77**	See Soofi 2012 above	Compared to the hand washing with antiseptic soap group, there was a 36% reduction in neonatal mortality in the CHX plus hand washing group (RR: 0.64, 95 % CI: 0.39–1.06), 26% reduction in the CHX only group (RR: 1.23, 95% CI: 0.50-1.08). Handwashing only had no effect on neonatal mortality (RR: 1.08, 95% CI: 0.79-1.48).<br>In a factorial analysis (CHX vs. no CHX), neonatal mortality was 38% lower in the

Table S1. Summaries of studies using randomized control trials (RCTs) to assess neonatal community-based interventions			
**Outcome measure**	**Author and year of publication**	**Population and study design**	**Results**
			CHX than the no CHX group (RR: 0.62, 95% CI: 0.45-0.85).
	Tripathy 2010 **S83**	Using a cluster-randomized design, this study aimed to assess the impact of facilitator-led women's groups on neonatal mortality for 19030 births over a 3 year period in two states in rural India.	NMR was 32% lower in intervention clusters (adjusted for clustering, stratification, and baseline differences) (OR 0.68, 95% CI 0.59-0.78) during the 3 years, and 45% lower in years 2 and 3 (0.55, 0.46-0.66).
Early neonatal mortality rate	Shankar 2008 **S73**	This double-blinded cluster-randomized trial implemented in Lombok, Indonesia, aimed to determine the effect of maternal multiple micronutrient supplementation on fetal loss and infant death. The study consisted of 262 clusters and enrolled 31,290 pregnant women. The intervention group received multiple micronutrients. Control group only received iron and folic acid (IFA).	Multiple micronutrients supplementation in pregnant women had no effect on early neonatal mortality (RR: 0.90, 95% CI: 0.76–1.06)
Perinatal mortality rate	Bang 2005 **S13**	See Bang 2005 above	Perinatal mortality rate in the intervention cluster was reduced by 56% (% change: 56, 95% CI: 44-68) between baseline and endline. Absolute difference in perinatal mortality rate between intervention and control group was 38 deaths per 1000 live births.
	Bhutta 2008 **S20**	See Bhutta 2008 above	Following the intervention, perinatal mortality rate in the intervention clusters at baseline decreased by 34.5 deaths per 1000 live births (P<0.05). In the control clusters, perinatal mortality rate increased by 6.9 deaths per 1000 live births between baseline and endline assessments.
	Colbourn 2013 **S24**	See Colbourn 2013 above	The odds of perinatal mortality was reduced by 16% in the clusters implementing both FI

Table S1. Summaries of studies using randomized control trials (RCTs) to assess neonatal community-based interventions			
**Outcome measure**	**Author and year of publication**	**Population and study design**	**Results**
			and CI interventions compared to the control clusters (OR:0.84, 95% CI:0-72-0.97)
	Jokhio 2005 **S39**	The study examined the effect of training traditional birth attendants and giving them clean disposable delivery kits on perinatal and maternal mortality in Pakistan. Three sub-districts were randomly assigned to an intervention group and four sub-districts to a control group. The study design was a cluster randomized controlled trial. 10,114 and 9184 pregnant women were enrolled in the study in the intervention and control sites, respectively.	The odds of perinatal death was 30% lower in the sub-districts with trained traditional birth attendants than the control sub-districts (Cluster-adjusted OR 0.70, 95% CI: 0.59-0.82).
	Kumar 2008 **S45**	See Kumar 2008 above	Compared to the control group, perinatal mortality was 42% lower in the essential newborn care arm (RR: 0.58, 95% CI: 0.44–0.77) and 33% lower in the essential newborn care plus ThermoSpot arm (RR: 0.67, 95% CI: 0.49–0.93).
	More 2012 **S52**	See More 2012 above	The stillbirth rate was non-significantly lower in the intervention arm (OR: 0.86, 95% CI: 0.60-1.22). Extended perinatal mortality did not differ between arms (OR: 1.19, 95% CI: 0.90-1.57).
	Shankar 2008 **S73**	See Shankar 2008 above	Multiple micronutrients supplementation in pregnant women had no effect on perinatal mortality (RR: 0.90, 95% CI: 0.79–1.03).

**Table S1. Summaries of studies using randomized control trials (RCTs) to assess neonatal community-based interventions**			
**Outcome measure**	**Author and year of publication**	**Population and study design**	**Results**
Infant mortality	Benn 2008 **S18**	Using data collected from a randomized-control trial of Vitamin A supplementation conducted in Guinea Bissau, the study tested the effect of oral polio vaccine on infant mortality. A total of 4345 normal birth weight infants were enrolled, 962 of which received oral polio vaccine (OPV).	The effect of OPV on infant mortality were different in boys and girls. Among boys, the risk of infant mortality was reduced by 63% (Mortality Rate Ratio: 0.37, 95% CI: 0.18-0.73), comparing those who received OPV at birth to those who did not. Among girls, receipt of OPV was not associated with infant mortality (Mortality Rate Ratio: 1.14, 95% CI: 0.70–1.89).
	Sloan 2008 **S76**	See Sloan 2008 above	Community-based kangaroo mother care had no effect on infant mortality (OR: 1.04, 95% CI: 0.77–1.40).
	Shankar 2008 **S73**	See Shankar 2008 above	Infant mortality was 18% lower in the multiple micronutrients group than the control group (RR: 0·82, 95% CI: 0·70–0·95).
Low birth weight-specific mortality rate	Sloan 2008 **S76**	See Sloan 2008 above	The odds of very low birth weight-specific mortality rate was reduced by 63%, comparing the community-based kangaroo mother care group to control clusters (OR: 0.37, 95% CI: 0.16–0.85).
	Tielsch 2007 **S81**	The study was carried out in Sarlahi District. A total of 8650 and 8880 live births were born in intervention and control clusters respective. The study was a community-based placebo-controlled, cluster randomized trial. After birth, newborns in the intervention group were cleansed with infant wipes that contained cholorhexidine.	Among newborns born low birth weight, there was a 28% reduction in neonatal mortality in the intervention relative to the control group (RR: 0.72, 96% CI: 0.55-0.95).
RR: Risk ratio or rate ratio<br>OR: Odds ratio<br>IRR: Incident rate ratio			

Appendix S2.

List of references for assessments included in the analysis for this paper

S1. Aga Khan Foundation/USA (2014). Chitral child survival project, Chitral, Khber Pakhtunkhwa, Pakistan: Final evaluation.

S2. Ahrari, M., Houser, R. F., Yassin, S., Mogheez, M., Hussaini, Y., Crump, P., . . . Levinson, F. J. (2006). A positive deviance-based antenatal nutrition project improves birth-weight in Upper Egypt. J Health Popul Nutr, 24(4), 498-507.

S3. Ali, M., Asefaw, T., Byass, P., Beyene, H., & Pedersen, F. K. (2005). Helping northern Ethiopian communities reduce childhood mortality: population-based intervention trial. Bull World Health Organ, 83(1), 27-33. doi:/S0042-96862005000100011

S4. Amano, S., Shrestha, B. P., Chaube, S. S., Higuchi, M., Manandhar, D. S., Osrin, D., . . . Saville, N. (2014). Effectiveness of female community health volunteers in the detection and management of low-birth-weight in Nepal. Rural Remote Health, 14(1), 2508.

S5. Anand, K., Kant, S., Kumar, G., & Kapoor, S. K. (2000). "Development" is not essential to reduce infant mortality rate in India: experience from the Ballabgarh project. J Epidemiol Community Health, 54(4), 247-253. doi:http://dx.doi.org/10.1136/jech.54.4.247

S6. Ansah Manu, A., ten Asbroek, A., Soremekun, S., Gyan, T., Weobong, B., Tawiah-Agyemang, C., . . . Kirkwood, B. R. (2014). Evaluating the implementation of community volunteer assessment and referral of sick babies: lessons learned from the Ghana Newhints home visits cluster randomized controlled trial. Health Policy Plan, 29 Suppl 2, ii114-127. doi:10.1093/heapol/czu080

S7. ASHA-India 2008. Overview of Asha/India.

S8. Awoonor-Williams, J. K., Feinglass, E. S., Tobey, R., Vaughan-Smith, M. N., Nyonator, F. K., & Jones, T. C. (2004). Bridging the gap between evidence-based innovation and national health-sector reform in Ghana. Stud Fam Plann, 35(3), 161-177. doi:http://dx.doi.org/10.1111/j.1728-4465.2004.00020.x

S9. Azad, K., Barnett, S., Banerjee, B., Shaha, S., Khan, K., Rego, A. R., . . . Costello, A. (2010). Effect of scaling up women's groups on birth outcomes in three rural districts in Bangladesh: a cluster-randomised controlled trial. Lancet, 375(9721), 1193-1202. doi:10.1016/S0140-6736(10)60142-0

S10. Balaluka, G. B., Nabugobe, P. S., Mitangala, P. N., Cobohwa, N. B., Schirvel, C., Dramaix, M. W., & Donnen, P. (2012). Community volunteers can improve breastfeeding among children under six months of age in the Democratic Republic of Congo crisis. Int Breastfeed J, 7, 2. doi:10.1186/1746-4358-7-2

S11. Bang, A. T., Bang, R. A., & Sontakke, P. G. (1994). Management of childhood pneumonia by traditional birth attendants. The SEARCH Team. Bull World Health Organ, 72(6), 897-905.

S12. Bang, A. T., Bang, R. A., Baitule, S. B., Reddy, M. H., & Deshmukh, M. D. (1999). Effect of home-based neonatal care and management of sepsis on neonatal mortality: field trial in rural India. Lancet, 354(9194), 1955-1961. doi:10.1016/S0140-6736(99)03046-9

S13. Bang, A. T., Reddy, H. M., Deshmukh, M. D., Baitule, S. B., & Bang, R. A. (2005). Neonatal and infant mortality in the ten years (1993 to 2003) of the Gadchiroli field trial: effect of home-based neonatal care. J Perinatol, 25 Suppl 1, S92-107. doi:10.1038/sj.jp.7211277

S14. Baqui, A. H., Arifeen, S. E., Williams, E. K., Ahmed, S., Mannan, I., Rahman, S. M., . . . Darmstadt, G. L. (2009). Effectiveness of home-based management of newborn

infections by community health workers in rural Bangladesh. Pediatr Infect Dis J, 28(4), 304-310. doi:10.1097/INF.0b013e31819069e8

S15. Baqui, A. H., Rosecrans, A. M., Williams, E. K., Agrawal, P. K., Ahmed, S., Darmstadt, G. L., . . . Santosham, M. (2008). NGO facilitation of a government community-based maternal and neonatal health programme in rural India: improvements in equity. Health Policy Plan, 23(4), 234-243. doi:10.1093/heapol/czn012

S16. Bashour, H. N., Kharouf, M. H., Abdulsalam, A. A., El Asmar, K., Tabbaa, M. A., & Cheikha, S. A. (2008). Effect of postnatal home visits on maternal/infant outcomes in Syria: a randomized controlled trial. Public Health Nurs, 25(2), 115-125. doi:10.1111/j.1525-1446.2008.00688.x

S17. Becker, S. R., Diop, F., & Thornton, J. N. (1993). Infant and child mortality in two counties of Liberia: results of a survey in 1988 and trends since 1984. Int J Epidemiol, 22 Suppl 1, S56-63. doi:http://dx.doi.org/10.1093/ije/22.supplement_1.s56

S18. Benn, C. S., Diness, B. R., Roth, A., Nante, E., Fisker, A. B., Lisse, I. M., . . . Aaby, P. (2008). Effect of 50,000 IU vitamin A given with BCG vaccine on mortality in infants in Guinea-Bissau: randomised placebo controlled trial. BMJ, 336(7658), 1416-1420. doi:10.1136/bmj.39542.509444.AE

S19. Bhandari, N., Mazumder, S., Taneja, S., Sommerfelt, H., Strand, T. A., & Group, I. E. S. (2012). Effect of implementation of Integrated Management of Neonatal and Childhood Illness (IMNCI) programme on neonatal and infant mortality: cluster randomised controlled trial. BMJ, 344, e1634. doi:10.1136/bmj.e1634

S20. Bhutta, Z. A., Soofi, S., Cousens, S., Mohammad, S., Memon, Z. A., Ali, I., . . . Martines, J. (2011). Improvement of perinatal and newborn care in rural Pakistan through community-based strategies: a cluster-randomised effectiveness trial. Lancet, 377(9763), 403-412. doi:10.1016/S0140-6736(10)62274-X

S21. Callaghan-Koru, J. A., Nonyane, B. A., Guenther, T., Sitrin, D., Ligowe, R., Chimbalanga, E., . . . Baqui, A. H. (2013). Contribution of community-based newborn health promotion to reducing inequities in healthy newborn care practices and knowledge: evidence of improvement from a three-district pilot program in Malawi. BMC Public Health, 13, 1052. doi:10.1186/1471-2458-13-1052

S22. Christian, P., Khatry, S. K., & West, K. P., Jr. (2004). Antenatal anthelmintic treatment, birthweight, and infant survival in rural Nepal. Lancet, 364(9438), 981-983. doi:10.1016/S0140-6736(04)17023-2

S23. Christian, P., Khatry, S. K., Katz, J., Pradhan, E. K., LeClerq, S. C., Shrestha, S. R., . . . West, K. P., Jr. (2003). Effects of alternative maternal micronutrient supplements on low birth weight in rural Nepal: double blind randomised community trial. BMJ, 326(7389), 571. doi:10.1136/bmj.326.7389.571

S24. Colbourn, T., Nambiar, B., Bondo, A., Makwenda, C., Tsetekani, E., Makonda-Ridley, A., . . . Costello, A. (2013). Effects of quality improvement in health facilities and community mobilization through women's groups on maternal, neonatal and perinatal mortality in three districts of Malawi: MaiKhanda, a cluster randomized controlled effectiveness trial. Int Health, 5(3), 180-195. doi:10.1093/inthealth/iht011

S25. Coutinho, S. B., de Lira, P. I., de Carvalho Lima, M., & Ashworth, A. (2005). Comparison of the effect of two systems for the promotion of exclusive breastfeeding. Lancet, 366(9491), 1094-1100. doi:10.1016/S0140-6736(05)67421-1

S26. Crookston, B. T., Dearden, K. A., Chan, K., Chan, T., & Stoker, D. D. (2007). Buddhist nuns on the move: an innovative approach to improving breastfeeding practices in Cambodia. Matern Child Nutr, 3(1), 10-24. doi:10.1111/j.1740-8709.2007.00074.x

S27. Datta, N., Kumar, V., Kumar, L., & Singhi, S. (1987). Application of case management to the control of acute respiratory infections in low-birth-weight infants: a feasibility study. Bull World Health Organ, 65(1), 77-82.

S28. Daulaire, N. M., Starbuck, E. S., Houston, R. M., Church, M. S., Stukel, T. A., & Pandey, M. R. (1992). Childhood mortality after a high dose of vitamin A in a high risk population. BMJ, 304(6821), 207-210. doi:http://dx.doi.org/10.1136/bmj.304.6821.207

S29. Dongre, A. R., Deshmukh, P. R., & Garg, B. S. (2009). A community based approach to improve health care seeking for newborn danger signs in rural Wardha, India. Indian J Pediatr, 76(1), 45-50. doi:10.1007/s12098-009-0028-y

S30. El Arifeen, S. E., Mullany, L. C., Shah, R., Mannan, I., Rahman, S. M., Talukder, M. R., . . . Baqui, A. H. (2012). The effect of cord cleansing with chlorhexidine on neonatal mortality in rural Bangladesh: a community-based, cluster-randomised trial. Lancet, 379(9820), 1022-1028. doi:10.1016/S0140-6736(11)61848-5

S31. el-Rafie, M., Hassouna, W. A., Hirschhorn, N., Loza, S., Miller, P., Nagaty, A., . . . Riyad, S. (1990). Effect of diarrhoeal disease control on infant and childhood mortality in Egypt. Report from the National Control of Diarrheal Diseases Project. Lancet, 335(8685), 334-338. doi:http://dx.doi.org/10.1016/0140-6736(90)90616-d

S32. Findley, S. E., Uwemedimo, O. T., Doctor, H. V., Green, C., Adamu, F., & Afenyadu, G. Y. (2013). Comparison of high- versus low-intensity community health worker intervention to promote newborn and child health in Northern Nigeria. Int J Womens Health, 5, 717-728. doi:10.2147/IJWH.S49785

S33. Fottrell, E., Azad, K., Kuddus, A., Younes, L., Shaha, S., Nahar, T., . . . Houweling, T. A. (2013). The effect of increased coverage of participatory women's groups on neonatal mortality in Bangladesh: A cluster randomized trial. JAMA Pediatr, 167(9), 816-825. doi:10.1001/jamapediatrics.2013.2534

S34. Gill, C. J., MacLeod, W. B., Phiri-Mazala, G., Guerina, N. G., Mirochnick, M., Knapp, A. B., & Hamer, D. H. (2014). Can traditional birth attendants be trained to accurately identify septic infants, initiate antibiotics, and refer in a rural African setting? Glob Health Sci Pract, 2(3), 318-327. doi:10.9745/GHSP-D-14-00045

S35. Gopinath, Rajani, MD, Consultant; Dipak P Tiwari, Care-Nepal; Nirmala Sharma, Care-Nepal; Raj Kahato, Care-Nepal; R Sharan Pyakurei, Care-Nepal; Khrist Roy, MD, Care Atlanta; Gundegmaa Jaamaa, MD, Care Atlanta; Melissa Larkin, Care Atlanta; Parini Shah, Care Atlanta. "Community Responsive Antenatal, Delivery, and Life Essential (CRADLE) Support Program (MANASHI): Doti and Kailali districts of Far Western Region of Nepal." December 31, 2011. Care – Nepal.

S36. Gupta, N., Cyamatare, F. R., Niyigena, P., Niyigena, J. W., Stulac, S., Mugwaneza, P., . . . Franke, M. F. (2013). Clinical outcomes of a comprehensive integrated program for HIV-exposed infants: a 3-year experience promoting HIV-free survival in rural Rwanda. J Acquir Immune Defic Syndr, 62(4), e109-114. doi:10.1097/QAI.0b013e31827d5118

S37. Haider, R., Ashworth, A., Kabir, I., & Huttly, S. R. (2000). Effect of community-based peer counsellors on exclusive breastfeeding practices in Dhaka, Bangladesh: a randomised controlled trial [see commments]. Lancet, 356(9242), 1643-1647. doi:http://dx.doi.org/10.1016/s0140-6736(00)03159-7

S38. Haider, R., Kabir, I., Huttly, S. R., & Ashworth, A. (2002). Training peer counselors to promote and support exclusive breastfeeding in Bangladesh. J Hum Lact, 18(1), 7-12. doi:http://dx.doi.org/10.1177/089033440201800102

S39. Jokhio, A. H., Winter, H. R., & Cheng, K. K. (2005). An intervention involving traditional birth attendants and perinatal and maternal mortality in Pakistan. N Engl J Med, 352(20), 2091-2099. doi:10.1056/NEJMsa042830

S40. Kagaayi, J., Dreyfuss, M. L., Kigozi, G., Chen, M. Z., Wabwire-Mangen, F., Serwadda, D., . . . Gray, R. H. (2005). Maternal self-medication and provision of nevirapine to newborns by women in Rakai, Uganda. J Acquir Immune Defic Syndr, 39(1), 121-124. doi:http://dx.doi.org/10.1097/01.qai.0000148530.66587.7c

S41. Khan, M. H., Khalique, N., Siddiqui, A. R., & Amir, A. (2013). Impact of behavior change communication among pregnant women regarding neonatal care. Indian J Pediatr, 80(10), 804-808. doi:10.1007/s12098-013-1076-x

S42. Khanal, S., Sharma, J., Gc, V. S., Dawson, P., Houston, R., Khadka, N., & Yengden, B. (2011). Community health workers can identify and manage possible infections in neonates and young infants: MINI--a model from Nepal. J Health Popul Nutr, 29(3), 255-264. doi:http://dx.doi.org/10.3329/jhpn.v29i3.7873

S43. Kimani-Murage, E. W., Norris, S. A., Mutua, M. K., Wekesah, F., Wanjohi, M., Muhia, N., . . . Griffiths, P. L. (2016). Potential effectiveness of Community Health Strategy to promote exclusive breastfeeding in urban poor settings in Nairobi, Kenya: a quasi-experimental study. J Dev Orig Health Dis, 7(2), 172-184. doi:10.1017/S2040174415007941

S44. Kirkwood, B. R., Manu, A., ten Asbroek, A. H., Soremekun, S., Weobong, B., Gyan, T., . . . Hill, Z. (2013). Effect of the Newhints home-visits intervention on neonatal mortality rate and care practices in Ghana: a cluster randomised controlled trial. Lancet, 381(9884), 2184-2192. doi:10.1016/S0140-6736(13)60095-1

S45. Kumar, V., Mohanty, S., Kumar, A., Misra, R. P., Santosham, M., Awasthi, S., . . . Saksham Study, G. (2008). Effect of community-based behaviour change management on neonatal mortality in Shivgarh, Uttar Pradesh, India: a cluster-randomised controlled trial. Lancet, 372(9644), 1151-1162. doi:10.1016/S0140-6736(08)61483-X

S46. Larocque, R., Casapia, M., Gotuzzo, E., MacLean, J. D., Soto, J. C., Rahme, E., & Gyorkos, T. W. (2006). A double-blind randomized controlled trial of antenatal mebendazole to reduce low birthweight in a hookworm-endemic area of Peru. Trop Med Int Health, 11(10), 1485-1495. doi:10.1111/j.1365-3156.2006.01706.x

S47. Lewycka S, Mwansambo C, Rosato M, Kazembe P, Phiri T, Mganga A, et al. Effect of women's groups and volunteer peer counselling on rates of mortality, morbidity, and health behaviours in mothers and children in rural Malawi (MaiMwana): a factorial, cluster-randomised controlled trial. Lancet. 2013;381:1721-35.

S48. Li, Y., Hotta, M., Shi, A., Li, Z., Yin, J., Guo, G., . . . Ushijima, H. (2007). Malnutrition improvement for infants under 18 months old of Dai minority in Luxi, China. Pediatr Int, 49(2), 273-279. doi:10.1111/j.1442-200X.2007.02349.x

S49. Malekafzali, H., Abdollahi, Z., Mafi, A., & Naghavi, M. (2000). Community-based nutritional intervention for reducing malnutrition among children under 5 years of age in the Islamic Republic of Iran. East Mediterr Health J, 6(2-3), 238-245.

S50. Manandhar, D. S., Osrin, D., Shrestha, B. P., Mesko, N., Morrison, J., Tumbahangphe, K. M., . . . Members of the MIRA Makwanpur trial team. (2004). Effect of a participatory intervention with women's groups on birth outcomes in Nepal: cluster-randomised controlled trial. Lancet, 364(9438), 970-979. doi:10.1016/S0140-6736(04)17021-9

S51. Memon, Z. A., Khan, G. N., Soofi, S. B., Baig, I. Y., & Bhutta, Z. A. (2015). Impact of a community-based perinatal and newborn preventive care package on perinatal and neonatal mortality in a remote mountainous district in Northern Pakistan. BMC Pregnancy Childbirth, 15, 106. doi:10.1186/s12884-015-0538-8

S52. More NS, Bapat U, Das S, Alcock G, Patil S, Porel M, et al. Community mobilization in Mumbai slums to improve perinatal care and outcomes: a cluster randomized controlled trial. PLoS Med. 2012;9:e1001257

S53. Mullany, L. C., Darmstadt, G. L., Khatry, S. K., Katz, J., LeClerq, S. C., Shrestha, S., . . . Tielsch, J. M. (2006). Topical applications of chlorhexidine to the umbilical cord for prevention of omphalitis and neonatal mortality in southern Nepal: a community-based, cluster-randomised trial. Lancet, 367(9514), 910-918. doi:10.1016/S0140-6736(06)68381-5

S54. Mullany, L. C., Shah, R., El Arifeen, S., Mannan, I., Winch, P. J., Hill, A., . . . Baqui, A. H. (2013). Chlorhexidine cleansing of the umbilical cord and separation time: a cluster-randomized trial. Pediatrics, 131(4), 708-715. doi:10.1542/peds.2012-2951

S55. Murray, John, Waltensperger, Karen Z., Marsh, David, Kabongo, John, Filumba, Stephen, Snetro, Gail, Lake-Post, Sharon (2014). Lufwanyama Integrated Neonatal and Child Health Project in Zambia (LINCHPIN): Final Evaluation. Building Community Systems to Improve the Health of Newborns and Children in Rural Zambia. Save the Children, Washington DC.

S56. Nalwadda, C. K., Waiswa, P., Kiguli, J., Namazzi, G., Namutamba, S., Tomson, G., . . . Guwatudde, D. (2013). High compliance with newborn community-to-facility referral in eastern Uganda:.an opportunity to improve newborn survival. PLoS One, 8(11), e81610. doi:10.1371/journal.pone.0081610

S57. Neumann, N. A., Victora, C. G., Halpern, R., Guimaraes, P. R., & Cesar, J. A. (1999). [Assessment of the performance of Pastoral de Crianca, a health support group, in promoting child survival and health education in Criciuma,a city in southern Brazil]. Rev Panam Salud Publica, 5(6), 400-410. doi:http://dx.doi.org/10.1590/s1020-49891999000500004

S58. Neutzling, M. B., Vieira, M. F., Cesar, J. A., Gigante, D. P., Martins, E. B., & Facchini, L. A. (1993). [Measuring the impact of promoting breastfeeding in primary health care services in Pelotas, Rio Grande do Sul, Brazil]. Cad Saude Publica, 9(2), 149-154. doi:/S0102-311X1993000200005

S59. Newell, K. W., Duenas Lehmann, A., LeBlanc, D. R., & Garces Osorio, N. (1966). The use of toxoid for the prevention of tetanus neonatorum. Final report of a double-blind controlled field trial. Bull World Health Organ, 35(6), 863-871.

S60. Orobaton, N., Abegunde, D., Shoretire, K., Abdulazeez, J., Fapohunda, B., Lamiri, G., . . . Osborne-Smith, M. (2015). A Report of At-Scale Distribution of Chlorhexidine Digluconate 7.1% Gel for Newborn Cord Care to 36,404 Newborns in Sokoto State, Nigeria: Initial Lessons Learned. PLoS One, 10(7), e0134040. doi:10.1371/journal.pone.0134040

S61. Osendarp, S. J., van Raaij, J. M., Darmstadt, G. L., Baqui, A. H., Hautvast, J. G., & Fuchs, G. J. (2001). Zinc supplementation during pregnancy and effects on growth and morbidity in low birthweight infants: a randomised placebo controlled trial. Lancet, 357(9262), 1080-1085. doi:http://dx.doi.org/10.1016/s0140-6736(00)04260-4

S62. Parashar, M., Singh, S., Kishore, J., Kumar, A., & Bhardwaj, M. (2013). Effect of Community-based Behavior Change Communication on Delivery and Newborn Health Care Practices in a Resettlement Colony of Delhi. Indian J Community Med, 38(1), 42-48. doi:10.4103/0970-0218.106627

S63. Penfold, S., Manzi, F., Mkumbo, E., Temu, S., Jaribu, J., Shamba, D. D., . . . Schellenberg, J. A. (2014). Effect of home-based counselling on newborn care practices in southern Tanzania one year after implementation: a cluster-randomised controlled trial. BMC Pediatr, 14, 187. doi:10.1186/1471-2431-14-187

S64. Perry, H., Cayemittes, M., Philippe, F., Dowell, D., Dortonne, J. R., Menager, H., . . . Berggren, G. (2006). Reducing under-five mortality through Hopital Albert Schweitzer's integrated system in Haiti. Health Policy Plan, 21(3), 217-230. doi:10.1093/heapol/czl005

S65. Perry HB, Valdez M, Stollak I, and Llanque R. (2016.) Focused Strategic Assessment: USAID Child Survival and Health Grants Program "Community-Based, Impact-Oriented Child Survival in Huehuetenango Guatemala." Curamericas Global, Inc. Raleigh, NC, USA.

S66. Persson, L. A., Nga, N. T., Malqvist, M., Thi Phuong Hoa, D., Eriksson, L., Wallin, L., . . . Ewald, U. (2013). Effect of Facilitation of Local Maternal-and-Newborn Stakeholder Groups on Neonatal Mortality: Cluster-Randomized Controlled Trial. PLoS Med, 10(5), e1001445. doi:10.1371/journal.pmed.1001445

S67. Qureshi, A. M., Oche, O. M., Sadiq, U. A., & Kabiru, S. (2011). Using community volunteers to promote exclusive breastfeeding in Sokoto State, Nigeria. Pan Afr Med J, 10, 8. doi:http://dx.doi.org/10.4314/pamj.v10i0.72215

S68. Rahman, M., Chen, L. C., Chakraborty, J., Yunus, M., Faruque, A. S., & Chowdhury, A. I. (1982). Use of tetanus toxoid for the prevention of neonatal tetanus. 2. Immunization acceptance among pregnant women in rural Bangladesh. Bull World Health Organ, 60(2), 269-277.

S69. Rana, S. B., Anand, H. (2011). "Local Innovation for Better Outcomes for Neonates (LIBON) Project. Final Evaluation Report." PLAN Nepal Child Survival Project XXII.

S70. Razzaque, A., Streatfield, P. K., & Gwatkin, D. R. (2007). Does health intervention improve socioeconomic inequalities of neonatal, infant and child mortality? Evidence from Matlab, Bangladesh. Int J Equity Health, 6, 4. doi:10.1186/1475-9276-6-4

S71. Rotheram-Borus, M. J., Tomlinson, M., le Roux, I. M., Harwood, J. M., Comulada, S., O'Connor, M. J., . . . Worthman, C. M. (2014). A cluster randomised controlled effectiveness trial evaluating perinatal home visiting among South African mothers/infants. PLoS One, 9(10), e105934. doi:10.1371/journal.pone.0105934

S72. Seim, A. R., Alassoum, Z., Bronzan, R. N., Mainassara, A. A., Jacobsen, J. L., & Gali, Y. A. (2014). Pilot community-mobilization program reduces maternal and perinatal mortality and prevents obstetric fistula in Niger. Int J Gynaecol Obstet, 127(3), 269-274. doi:10.1016/j.ijgo.2014.06.016

S73. Shankar, A. H., Jahari, A. B., Sebayang, S. K., Aditiawarman, Apriatni, M., . . . Sofia, G S86 Supplementation with Multiple Micronutrients Intervention Trial (SUMMIT) Study Group, (2008). Effect of maternal multiple micronutrient supplementation on fetal loss and infant death in Indonesia: a double-blind cluster-randomised trial. Lancet, 371(9608), 215-227. doi:10.1016/S0140-6736(08)60133-6

S74. Singh, K., Brodish, P., & Haney, E. (2014). Postnatal care by provider type and neonatal death in sub-Saharan Africa: a multilevel analysis. BMC Public Health, 14, 941. doi:10.1186/1471-2458-14-941

S75. Sitrin, D., Guenther, T., Waiswa, P., Namutamba, S., Namazzi, G., Sharma, S., . . . Moran, A. (2015). Improving newborn care practices through home visits: lessons from Malawi, Nepal, Bangladesh, and Uganda. Glob Health Action, 8, 23963. doi:10.3402/gha.v8.23963

S76. Sloan, N. L., Ahmed, S., Mitra, S. N., Choudhury, N., Chowdhury, M., Rob, U., & Winikoff, B. (2008). Community-based kangaroo mother care to prevent neonatal and infant mortality: a randomized, controlled cluster trial. Pediatrics, 121(5), e1047-1059. doi:10.1542/peds.2007-0076

S77. Soofi, S., Cousens, S., Imdad, A., Bhutto, N., Ali, N., & Bhutta, Z. A. (2012). Topical application of chlorhexidine to neonatal umbilical cords for prevention of omphalitis and neonatal mortality in a rural district of Pakistan: a community-based, cluster-randomised trial. Lancet, 379(9820), 1029-1036. doi:10.1016/S0140-6736(11)61877-1

S78. Spencer, H. C., Kaseje, D. C., Mosley, W. H., Sempebwa, E. K., Huong, A. Y., & Roberts, J. M. (1987). Impact on mortality and fertility of a community-based malaria control programme in Saradidi, Kenya. Ann Trop Med Parasitol, 81 Suppl 1, 36-45.

S79. Syed, U., Asiruddin, S., Helal, M. S., Mannan, II, & Murray, J. (2006). Immediate and early postnatal care for mothers and newborns in rural Bangladesh. J Health Popul Nutr, 24(4), 508-518.

S80. Thiam, L., & World Vision/Senegal (1994, Oct 2-7, 1994). Impact of the Thies CSP on the Health Knowledge and Practices of Mothers Living in the Sub-district of Niakhene (Thies region). Paper presented at the Community Impact of PVO Child Survival Efforts: 1995 - 1994, Bangalore, Karnataka, India.

S81. Tielsch, J. M., Darmstadt, G. L., Mullany, L. C., Khatry, S. K., Katz, J., LeClerq, S. C., . . . Adhikari, R. (2007). Impact of newborn skin-cleansing with chlorhexidine on neonatal mortality in southern Nepal: a community-based, cluster-randomized trial. Pediatrics, 119(2), e330-340. doi:10.1542/peds.2006-1192

S82. Tielsch, J. M., Rahmathullah, L., Katz, J., Thulasiraj, R. D., Coles, C., Sheeladevi, S., & Prakash, K. (2008). Maternal night blindness during pregnancy is associated with low birthweight, morbidity, and poor growth in South India. J Nutr, 138(4), 787-792.

S83. Tripathy, P., Nair, N., Barnett, S., Mahapatra, R., Borghi, J., Rath, S., . . . Costello, A. (2010). Effect of a participatory intervention with women's groups on birth outcomes and maternal depression in Jharkhand and Orissa, India: a cluster-randomised controlled trial. Lancet, 375(9721), 1182-1192. doi:10.1016/S0140-6736(09)62042-0

S84. Uzondu, C. A., Doctor, H. V., Findley, S. E., Afenyadu, G. Y., & Ager, A. (2015). Female health workers at the doorstep: a pilot of community-based maternal, newborn, and child health service delivery in northern Nigeria. Glob Health Sci Pract, 3(1), 97-108. doi:10.9745/GHSP-D-14-00117

S85. Vir, S. C. (2013). Community based maternal and child health nutrition project, uttar pradesh: an innovative strategy focusing on "at risk" families. Indian J Community Med, 38(4), 234-239. doi:10.4103/0970-0218.120159

S86. Vogt, F., Ferreyra, C., Bernasconi, A., Ncube, L., Taziwa, F., Marange, W., . . . Becher, H. (2015). Tracing defaulters in HIV prevention of mother-to-child transmission programmes through community health workers: results from a rural setting in Zimbabwe. J Int AIDS Soc, 18, 20022. doi:10.7448/IAS.18.1.20022

S87. Waiswa, P., Pariyo, G., Kallander, K., Akuze, J., Namazzi, G., Ekirapa-Kiracho, E., . . . Uganda Newborn Study, T. (2015). Effect of the Uganda Newborn Study on care-seeking and care practices: a cluster-randomised controlled trial. Glob Health Action, 8, 24584. doi:10.3402/gha.v8.24584

S88. Wangalwa, G., Cudjoe, B., Wamalwa, D., Machira, Y., Ofware, P., Ndirangu, M., & Ilako, F. (2012). Effectiveness of Kenya's Community Health Strategy in delivering community-based maternal and newborn health care in Busia County, Kenya: non-randomized pre-test post test study. Pan Afr Med J, 13 Suppl 1, 12.
